# Pilot Educational Intervention and Feasibility Assessment of Breast Ultrasound in Rural South Africa

**DOI:** 10.1200/JGO.2016.008086

**Published:** 2017-03-27

**Authors:** Lindsay K. Dickerson, Anne F. Rositch, Susan Lucas, Susan C. Harvey

**Affiliations:** **Lindsay K. Dickerson**, Johns Hopkins University School of Medicine; **Anne F. Rositch**, Johns Hopkins Bloomberg School of Public Health; **Susan C. Harvey**, Johns Hopkins Medical Institutions, Baltimore, MD; and **Susan Lucas**, Chris Hani Baragwanath Academic Hospital, University of Witwatersrand School of Medicine, Johannesburg, South Africa.

## Abstract

**Purpose:**

Breast cancer is the leading cause of cancer death in women worldwide, with high mortality in low- and middle-income countries because of a lack of detection, diagnosis, and treatment. With mammography unavailable, ultrasound offers an alternative for downstaging. The literature reports successful training in various domains, but a focus on the breast is novel. We assessed the feasibility (knowledge acquisition, perceived usefulness, and self-efficacy) of breast ultrasound training for nonphysician providers.

**Methods:**

Training was implemented for 12 providers at Hlokomela Clinic in Hoedspruit, South Africa, over 3 weeks. Didactic presentations and example cases were followed by a presurvey and test (n = 12). All providers received hands-on training with nurses as models; five providers trained with patients. A post-test (n = 12) assessed knowledge acquisition and a postsurvey (n = 10) assessed perceived program usefulness and provider self-efficacy.

**Results:**

The pre- to post-test averages improved by 68% in total and in four competencies (foundational knowledge, descriptive categories, benign *v* malignant, and lesion identification). On the postsurvey, providers expressed that ultrasound could significantly influence breast cancer detection (9.1 out of 10), treatment (7.9 out of 10), and survival (8.7 out of 10) in their community and endorsed moderate confidence in their scanning (6.3 out of 10) and interpreting abilities (5.6 out of 10).

**Conclusion:**

Our research supports the feasibility of breast ultrasound training as part of a breast education program in low- and middle-income countries. Pre- and post-test results and observed proficiency indicate that training nonphysician providers is achievable; postsurvey responses indicate program acceptance, community-based ownership, and provider self-efficacy with ultrasound. Future work may show that breast ultrasound is viable for early detection where mammography is unavailable.

## INTRODUCTION

Breast cancer, the leading cause of cancer death among women,^1^ is becoming an increasingly urgent problem in low- and middle-income countries (LMICs). By 2020, more than 1 million cases per year are projected to occur in LMICs alone,^2^ representing 70% of all cases worldwide.^3^ Although mortality rates in developed countries have decreased,^4^ they remain disproportionately high in LMICs because of late-stage presentation, indicating barriers in early detection and scarcity of resources for optimal treatment.^5,6^ For example, in a South African report, 78% of black women who had cancer presented with advanced-stage disease,^7^ which is consistent with a disparate 5-year survival rate of 53% in South Africa compared with 89% in the United States between 2005 and 2009.^1^ In addition, lack of awareness about breast cancer and screening poses barriers to downstaging.^8,9^ Strong evidence supports that early-stage diagnosis allows for initiation of effective treatment, which is vital to improving outcomes in LMICs.^8,10,11^

Although mammography is the gold standard for screening in developed countries, it is currently infeasible in limited-resource settings.^10,11^ Resources, infrastructure, and access to skilled breast care teams determine which screening tool is best for each location, from clinical breast examination (CBE) to mammography and molecular breast imaging.^8,12^ Ultrasound, used in developed countries to augment mammography and to examine localized findings, offers a viable alternative for screening in LMICs given the technology’s economy, portability, and versatility.^11,13^ Breast ultrasound has been shown to be particularly useful for imaging palpable lesions, differentiating cystic and solid masses, and describing lesion features, thus aiding in the assessment of the likelihood of malignancy.^11,14^ Moreover, it has been argued that the breast cancer detection rate with ultrasound is comparable to the detection rate with mammography,^15,16^ and potentially greater than mammography in women with dense breasts.^17^ However, high false-positive rates have been put forth as a potential drawback of ultrasound screening.^18^ Although the literature reports successful training and use of portable ultrasound devices in limited-resource settings across many domains, a multiweek curriculum focused on breast ultrasound training for nonphysician providers is novel.^19-21^

Our study assessed the feasibility, primarily defined by knowledge acquisition, perceived usefulness, and provider self-efficacy, of a breast ultrasound training program for nonphysician providers. Incorporated into an integrated early detection and education program in limited-resource settings, breast ultrasound has the potential to improve breast lesion characterization and thus enhance detection at stages when treatment is more effective, with the ultimate goal of reducing breast cancer mortality.

## METHODS

### Study Design and Setting

Our pilot study assessed the feasibility of training nonphysician providers (n = 12) in a limited-resource setting to use ultrasound for breast lesion detection. The curriculum-based training program included learning objectives, experiential hands-on training, and both pre- and posteducational assessments and was implemented at Hlokomela Clinic in Hoedspruit, South Africa. The clinic serves farm workers in the Maruleng and Bushbuckridge municipalities in the Limpopo and Mpumalanga provinces, respectively. The site was chosen for its nonprofit status, prominent standing in the community, and the staff’s willingness to incorporate breast cancer care into services offered. Training was focused on nonphysician providers because of their paramount role in patient care at Hlokomela and because of our belief that this would be the most sustainable approach.

### Ultrasound Educational Intervention

The 3-week training program began with introductory didactic presentations that included breast cancer facts, indications for ultrasound, breast anatomy, ultrasound technical factors, lesion characterization, and example cases. The presentations were modified versions of presentations developed by a team with experience in implementing similar education programs in low-resource areas,^21^ and were based on the American College of Radiology Breast Imaging Reporting and Data System (ACR BI-RADS) Atlas.^22^ The introductory session was followed by a presurvey (n = 12) to assess providers’ initial attitudes toward early detection and breast cancer knowledge. A pretest (n = 12; Data Supplement) was administered to elucidate areas in which to focus training and to serve as a baseline score for post-training comparison. Additional didactic resources were provided throughout the training, including handouts summarizing critical teaching points and step-by-step instructions for using the ultrasound equipment (Data Supplement), the ACR BI-RADS Atlas, and case discussions.

Most of the program consisted of experiential training. All providers received hands-on ultrasound training with nurses acting as models. Among the 12 providers, training was increased systematically so that five providers had additional training with 21 patients in total. Each patient visit involved gathering personal and family histories of breast cancer; screening for common symptoms including lump or thickening, discharge, skin changes, pain, and nipple abnormalities; performing a CBE; counseling on breast self-examination technique; and conducting whole breast ultrasound with the Chison Portable Eco 3, Version 1.0. Providers first observed patient visits, but by the program’s end were conducting visits under the supervision of the on-site trainer. This trainer performed a repeat CBE to ensure accuracy and reviewed scanning in real time. Images of any concerning ultrasound findings were sent to breast imaging radiologists (S.L., S.C.H.) for prompt consultation.

On-site ultrasound training, including the introductory presentations, was conducted by a medical student who had undergone an intensive 5-month breast ultrasound training program at Johns Hopkins Radiology in Baltimore, MD (L.K.D.). Breast imaging radiologists (S.L., S.C.H.) were involved extensively in all aspects of the training, including ensuring that the medical student was proficient in breast ultrasound, vetting the program curriculum and all training materials, communicating regularly during program implementation, and being readily available to provide remote consultation for ultrasound images. In addition, a radiologist (S.L.) visited Hlokomela before training, to ensure everything was in place for training to begin and to teach CBE to Hlokomela’s staff, and after training completion (S.C.H.), to assess the program’s success and to collaborate with Hlokomela on follow-up and future plans.

### Assessment

Two methods of assessment were used to evaluate the training. First, a post-test (n = 12; Data Supplement) assessed knowledge retention and acquisition. The pre- and post-test content, which was based on the didactic presentations, ACR BI-RADS Atlas, and learning objectives, evaluated four competencies: foundational knowledge, descriptive categories for masses, benign and malignant characteristics, and lesion identification. Second, a postsurvey (n = 10) assessed program acceptance, perceived ultrasound usefulness, perceived successes and limitations of training, and provider self-efficacy and investment. Furthermore, provider proficiency with ultrasound and in conducting visits was observed by the on-site trainer.

### Analysis

Pre- and post-test averages with 95% CIs and the percentage increase in test averages (n = 12) were calculated for the four competencies and the total scores. Paired *t* tests were used to determine if the difference between the pre- and post-test scores was significant (α = 0.05). Pre- and post-test averages and percentage increases were also calculated for the five providers who had undergone additional training. Average ratings on a 1 to 10 scale for postsurvey responses were calculated (n = 10 and n = 5). Provider responses regarding areas in which they felt most and least confident after training were summarized in pie charts (providers could list as many areas as desired).

## RESULTS

A total of 12 nonphysician providers, including nurses (n = 4), nursing assistants (n = 3), and lay counselors (n = 5), completed the program. On the presurvey, all 12 providers responded that early detection is very important for survival (mean = 5, out of 5 possible), that breast ultrasound can detect cancer earlier, and that patients would be willing to receive breast ultrasound. Answers were more varied with respect to knowledge about the lifetime chance of developing breast cancer and 5-year survival in South Africa: 75% of providers correctly identified the answer to the former, whereas 33% correctly identified the latter.

The pre- to post-test total averages (n = 12) improved by 68%, from 12.3 out of 28 points on the pretest to 20.8 out of 28 points on the post-test, with slightly greater increases for the five providers with additional patient training (71% improvement). Averages improved in all four competencies—foundational knowledge (focal zone placement, normal breast anatomy, and breast cancer symptoms); descriptive categories for mass characterization on the basis of the ACR BI-RADS lexicon (shape, orientation, margin, echo pattern, posterior features); benign and malignant characteristics; and lesion identification of common breast and axillary findings—by 59%, 72%, 28%, and 125%, respectively (*P* value for test of difference < .01, except for the “benign *v* malignant” competency *P* = .14; Table 1).

**Table 1:**
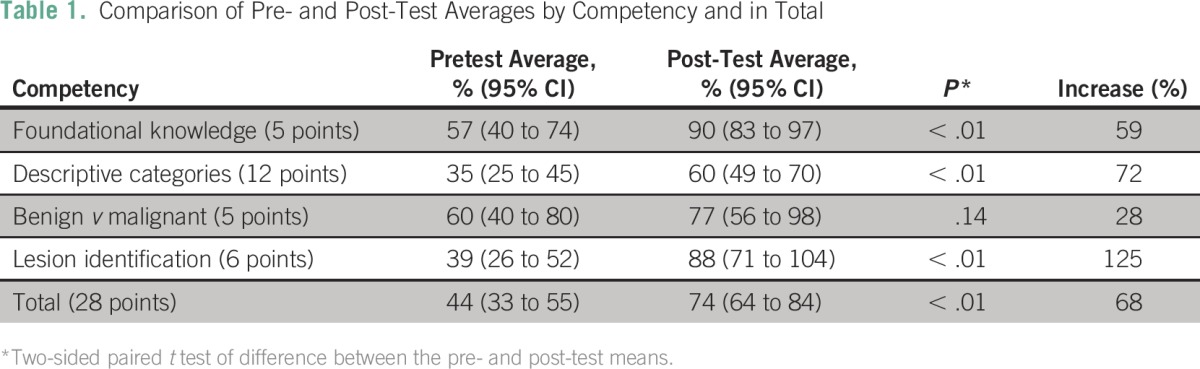
Comparison of Pre- and Post-Test Averages by Competency and in Total

After the educational intervention, providers recognized that ultrasound could have a significant impact on breast cancer detection, treatment, and survival in their community, with an average rating on a 1 to 10 scale of 9.1, 7.9, and 8.7, respectively. There was a trend for higher ratings among the five with more training (9.6, 9.2, and 9.6, respectively). Providers also viewed the training program as useful (7.9 out of 10 [n = 10] and 9 out of 10 [n = 5]) and enjoyable (8.8 out of 10 [n = 10] and 10 out of 10 [n = 5]). Moreover, they indicated considerable investment in continuing breast ultrasound (7.5 out of 10 [n = 10] and 8.2 out of 10 [n = 5]) and spreading breast cancer awareness (9.3 out of 10 [n = 10] and 9.4 out of 10 [n = 5]; Table 2).

**Table 2:**
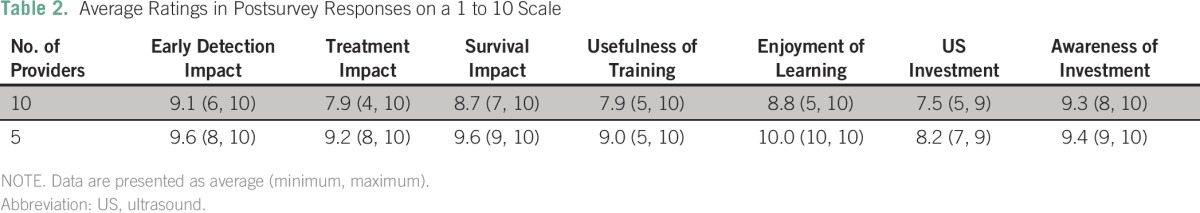
Average Ratings in Postsurvey Responses on a 1 to 10 Scale

**Fig 1:**
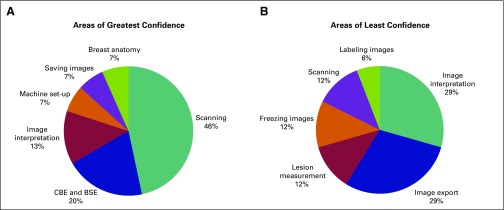
(A) Areas in which providers expressed greatest confidence (average confidence in scanning, 6.3 out of 10). (B) Areas in which providers expressed least confidence (average confidence in image interpretation, 5.6 out of 10). BSE, breast self-examination; CBE, clinical breast examination.

Providers expressed moderate confidence in scanning (6.3 out of 10) and image interpretation (5.6 out of 10), with the five with more training indicating slightly greater assurance (6.4 and 6.2, respectively). When asked to comment, providers listed scanning (“moving the [ultrasound] probe”), performing a CBE (“palpation”), and breast self-examination counseling as the skills in which they felt most confident (Fig 1A). However, they endorsed the need for additional practice to gain more self-assurance in image interpretation, image export (especially the process of sending images for remote consultation), and technical aspects such as measuring lesions and freezing and labeling images (Fig 1B).

## DISCUSSION

In this pilot study, we implemented a short-term educational intervention involving didactic and experiential components that trained and assessed nonphysician providers in the use of breast ultrasound. We found that training for only 3 weeks resulted in acquisition of knowledge and skills, as well as provider self-efficacy. Furthermore, the intervention was well received, rated highly for usefulness, and successful in promoting community-based ownership of breast cancer awareness. Our research supports the feasibility of a training program for use of breast ultrasound in limited-resource settings as part of a larger breast cancer detection and education campaign.

Significant improvement from pre- to post-test scores in all four competencies indicates that trainees can learn practical information about breast ultrasound, thus demonstrating proficient knowledge after a short training course. This is consistent with the findings of a recent review of the use of portable ultrasound in LMICs by Becker et al,^23^ which suggested that short training programs, even for trainees with limited prior experience, can lead to substantial knowledge retention and skill acquisition. We consider the pre- to post-test objective assessment of knowledge acquisition to be a strength of our study.

We recognize that successful and sustainable education programs necessitate an affirmation of the local relevance of the project. To this end, Morgan and Deutschmann^24^ maintain that effective training programs in LMICs must maximize learner input and stake. Postsurvey responses indicate that providers felt strongly about breast ultrasound’s usefulness, with one commenting that its role is “early detection leading to early [treatment] and expanding life to the community.” In addition to viewing breast ultrasound as a valuable local service, providers demonstrated a commitment both to incorporating ultrasound into clinic practice and to promoting breast cancer awareness. One provider expressed the desire to “go to the women’s ministry and give a health talk about my experience and knowledge.” Thus, we conclude that breast ultrasound training was well received and may become a point of care as providers use the visit for patient education, and interpret this as a successful first step in building a sustainable program.

Providers expressed self-efficacy in skills fundamental to a breast ultrasound visit such as conducting scans and CBE. Although providers’ average confidence ratings were moderate, the on-site trainer observed a level of proficiency that would allow providers to perform independently with continued support. We view the providers’ desire for additional practice as a commitment to learning and skill mastery. Moreover, we systematically increased training among the 12 providers to try to understand how the magnitude of training might influence post-test scores, confidence, and opinions about breast ultrasound usefulness. We noted that the five providers who received additional training had slightly greater improvements in pre- to post-test scores and gave higher average ratings across all postsurvey questions. Although we can generally interpret this to mean that increased training correlates with greater knowledge and skills acquisition, statistically significant comparisons are limited because of the small sample size.

As noted across numerous studies, ultrasound has many advantages as a diagnostic imaging modality because it is safe, portable, inexpensive, and versatile; requires minimal maintenance; and is relatively easy to learn.^11,19,20,23^ Ultrasound continues to gain widespread use in limited-resource settings,^13,19-21,25^ with applications in domains such as emergency medicine, obstetrics, cardiology, and infectious diseases.^19,26,27^ Furthermore, a number of studies have demonstrated success in ultrasound training exclusively for nonphysician providers.^28-31^ With breast cancer incidence and mortality rising steeply,^3,5,6^ we agree with Yip et al^6^ that research in LMICs should focus on strategies to downstage breast cancer at presentation. In addition, with mammography currently infeasible^10,11^ and a general shortage of doctors in limited-resource and rural settings,^32,33^ we believe breast ultrasound could fill a technology and physician gap in early breast cancer detection. In a recent breast ultrasound pilot project in the Kamuli District of Uganda, an experienced local sonographer was trained over 8 days in breast ultrasound, and all images were sent to an American board-certified radiologist for review. The authors concluded that breast ultrasound is a resource-appropriate strategy for breast cancer downstaging in LMICs.^34^ Our work offers a fresh perspective because it involved a multiweek training program in both breast ultrasound use and image interpretation, was designed for local providers with no prior imaging experience, and relied on remote radiologists for occasional image consultation only.

A limitation of our study was that, although most providers spoke English well, there was a minor language barrier with less common words or more complicated medical terminology. This may have contributed to difficulty in communicating certain nuances of the pre- and post-test and survey questions. However, our data confirm that most of the training materials and assessments were well understood. Furthermore, because of the narrow timeframe, we could not systematically assess the trainees’ ability to properly and consistently detect lesions. We agree with the point made by Adler et al^20(p265)^ after the introduction of a portable ultrasound into a Tanzanian refugee camp that objectively assessing trainee skills is critical to knowing that ultrasound is being used “effectively in medical decision making.” We also agree with the conclusion made by Lagrone et al^26^ in their review of ultrasound training opportunities that maintaining connections with local implementers is invaluable for long-term training success.

To this end, a radiologist (S.L.) has begun monthly visits to Hlokomela to continue ultrasound training and ensure quality control. This also addresses another potential limitation of our study—that time and travel constraints did not allow for board-certified radiologists to be on site during the 3-week training program. Because this was a feasibility assessment, our intention was to ascertain whether implementing a breast ultrasound training program for providers in limited-resource settings is achievable and thus has the potential to be beneficial with the direct involvement of breast imaging experts and longitudinal follow-up. Building on the success of this pilot study, future research will assess the efficacy of breast ultrasound screening by nonphysician providers in a longitudinal clinical study overseen by qualified medical professionals and with appropriate quality control measures.

The use of ultrasound in LMICs is well established, as is the efficacy of short-term training programs when post-training quality assessment and continued support are provided. Our pilot study is unique in that it involves breast ultrasound as a way to address the escalating urgency of breast cancer care by primarily nonphysician providers in limited-resource settings. Our findings support the feasibility of breast ultrasound training in rural South Africa, with the larger implication being that breast ultrasound could become a viable downstaging tool and point of care in other limited-resource settings where mammography is unavailable. Future directions should focus on obtaining additional high-quality data on the effectiveness and cost of ultrasound as a breast cancer screening tool in LMICs, including consideration of its potential limitations. Furthermore, we acknowledge that any early-detection technique must be paired with accessible diagnosis and treatment, work that we are exploring through additional projects. The vision is one of available, accessible, and centralized breast cancer care for women globally, empowering women to seek breast care early when lives can be saved by effective treatment. Next steps include needs and readiness assessment for larger populations and ultimately, implementation on a greater scale.
